# Understanding metro station areas’ functional characteristics via embedding representation: A case study of shanghai

**DOI:** 10.1038/s41598-025-87336-6

**Published:** 2025-01-21

**Authors:** Heping Jiang, Ruihua Liu, Shijia Luo, Disheng Yi, Jing Zhang

**Affiliations:** 1 Chengdu Center, China Geological Survey (Southwest China Center for Geoscience Innovation), Chengdu, 610218 China; 2 The High School Affiliated to Beijing International Studies University, Beijing, 100024 China; 3 Beijing Guangqumen Middle School, Beijing, 100062 China; 4 College of Resources Environment and Tourism, Capital Normal University, Beijing, 100048 China

**Keywords:** Metro station area, Functional characteristics, Text representation, POI data, Environmental sciences, Sustainability, Environmental impact

## Abstract

As crucial transportation hubs for urban travel, metro stations catalyze the transformation of their surrounding areas into highly prominent locations where many activities converge. Uncovering the functional attributes of station areas holds immense significance in comprehending citizens’ activity demands, thereby offering valuable insights for regional development and planning in proximity to metro stations. This study introduces a framework that improves the process of accurately representing station areas. On the basis of the semantic vectors of point of interests (POI) categories trained by the GloVe model, the partition smooth inverse frequency (P-SIF) model and affinity propagation (AP) are employed to generate the embedding representations of station areas and categorize. Finally, we classify the station areas into 9 functional groups: and analyse the spatial distribution characteristics of each group. It is found that most of the station areas in Shanghai show the characteristics of mixed type, in which the characteristics of residential type and commercial type are obvious. In terms of spatial, the stations with commercial characteristics are mainly distributed in the central area of the city, while those with residential and working characteristics are scattered.

## Introduction

In cities, metro stations are important transportation nodes that carry many intracity trips and constitute an important part of the urban spatial structure^1,2^. The station areas serve as destination hubs, attracting a multitude of socioeconomic activities and enjoying a high level of recognition^3,4^. Station areas usually cover high-density enterprises, residential areas, and various public facilities. Thus, these areas that serve the economic, leisure, and living needs of urban residents are considered special urban spaces with rich urban functions^5^. The different configurations of facilities in the station areas cause the stations to have different functional characteristics and play different roles^6^. Exploring the functions of metro station areas is helpful for research on forecasting metro passenger flow^7–9^, discovering travel patterns^10,11^, identifying the spatial structure of urban functions^12,13^, which further provides references for metro operation management and organization, the construction and development of station areas, and the realization of a balanced urban function structure.

Relevant studies on identifying the functional characteristics of station areas have usually been conducted from two perspectives: the mobility and the place features of station areas^14–18^. The former is mainly based on travel patterns extracted from mobility datasets, such as smart card data, which infers the potential functional characteristics of the stations according to the spatiotemporal regularity characteristics of residents’ travel behavior^19^. The latter usually classify stations into different functional types, mostly by relying on information on land use or the configuration of the POI in the station areas^18,20,21^. POI datasets are spatial vector point data with precise coordinates that record the type and semantic information of geographic entities. Compared with traditional land use data, POI data can describe the functional use of urban places at a finer scale^8,22,23^ and play an important role in research on the urban regional environment and functional structure^6,11,24,25^. The station areas usually cover a small area, and the land use data within the station areas is usually simple, which can not provide enough information about human activities. More importantly, a POI serves as a destination for people traveling to visit by subway^11,26^ and reflects the functional and travel interpretation of the metro station area. Therefore, we can infer the functional characteristics of the station area according to the spatial configuration and distribution relationship of different types of POIs in the station area space^5,6,11,24^.

Many methods have been proposed to describe the functional characteristics of station areas via POIs. Statistical-based methods measure the functional characteristics of a station area by calculating the proportion or distribution density of different types of POI^14,27,28^. Considering that the potential information of a POI is not fully mined through simple statistics, some researchers refer to the idea of topic extraction from text mining in the field of natural language processing (NLP) and adopt an analogy strategy from urban elements to text materials. They regard the station area as a document, the POI in the area as a word, and the potential functional semantic information in the regional POI datasets as the topic in the document. Therefore, many text mining methods in NLP are widely used, such as term frequency-inverse document frequency (TF-IDF)^17,24^ and latent Dirichlet allocation (LDA)^6,15^. However, owing to the limited number of stations, LDA methods do not seem to be efficient in understanding the semantics of stations^17^. These methods rely mainly on the frequency or probability information of the POIs and lose the spatial distribution information of the POIs.

To overcome such limitations, embedding methods are effective for discovering the utilization of urban regions^29–32^. The embedding methods treat the spatial arrangement order of the POIs in the region as the grammatical order of the words in the sentence and train the vector representations of the POIs in the semantic space on the basis of the contextual relationship of the spatial arrangement of the POIs. Then, the vectors of urban areas are computed on the basis of the local combination of different POIs and used to identify urban functional types^29^. There are many examples of embedding methods. The word2vec embedding model was first introduced for training the vector representations of POIs in the functional zoning problem^30^. The Place2vec model trains a neighborhood corpus of POI according to Tobler’s first law^31^. The topic word embedding (TWE) model considers the homonymy and polysemy issues of POIs in urban space and trains to obtain POI embedding vectors with topic feature information^32^. The global context window (GloVe) embedding model combines the advantages of global matrix factorization and local context window methods, integrating global statistical information based on the local context window approach, which can outperform related models on similarity tasks in NLP. Studies of urban functional region identification have adopted models to extract the spatial semantic information of POIs and have verified their validity^33,34^.

Importantly, these neural network embedding models only train vectors for POI categories, meaning that we have to calculate compound vectors to represent station areas on the basis of the POI vectors for later identification of station areas through clustering. Previous studies used the simple average summation of the vectors of POIs within areas to calculate the vectors of urban areas. These aggregating processes fail to capture the thematic heterogeneity among POI categories. Station areas often attach high-density POIs that belong to different functional topics^6^. In station areas, complex and various functional regions are formed around metro stations^18^. Calculating the vectors of station areas from simple averaging of POIs ignores topical information and captures fewer semantic meanings of long text. However, studies on how to integrate POI spatial semantic information to accurately represent station areas are still insufficient. In this study, we integrated the P-SIF document embedding method and POI data to obtain a vector representation of the station area and explore the functional features of the station areas. The P-SIF embedding method, which is a partitioned word averaging model to represent long documents^35^, can consider the importance and topic information of POIs within station areas in the process of aggregating the POI vectors to area vectors, assisting in more accurate representation and identification of the station areas.

The main contribution of this paper is to propose a research framework for accurately representing the semantics of station area using POI embedding vectors. In this framework, GloVe model training comprehensively considers the global and local POI information, learns the relationship between POI according to the co-occurrence probability of POI, and generates semantic vectors of different POI types. Compared with other commonly used methods, P-SIF takes into account the topic information of POI to obtain accurate semantic vectors of the station area when aggregating POI semantic vectors. The combination of the two methods can obtain more detailed and accurate functional characteristics of station areas. This paper is structured as follows. Section**‘‘Study area and materials’’** describes the study area and dataset. Section**‘‘Study area’’** introduces the framework process and methods of the research, including the training method of POI type vectors, the generation method of station area embedding vectors, and the clustering and analysis methods. Section**‘‘Data and preprocessing’’** describes the analysis results of the functional characteristics of the metro station area. Section**‘‘Methodology’’** compares the P-SIF method with existing integration methods. Section**‘‘Methodology’’** summarizes and discusses the findings, implications, and limitations of this research, as well as some ideas for future research.

## Study area and materials

### Study area

**Fig. 1:**
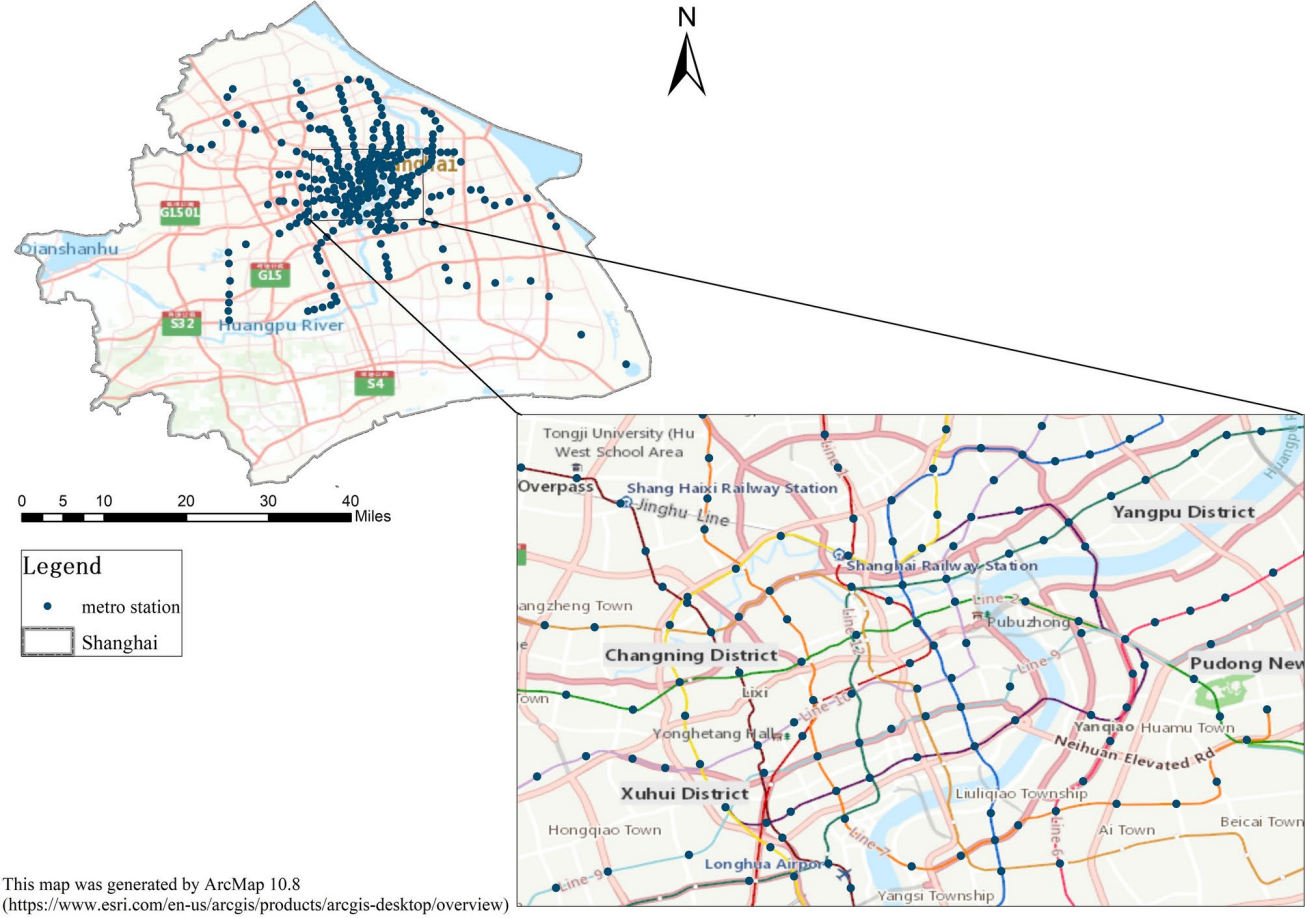
Study area and metro distribution. Global spatial distribution of Shanghai metro stations and local distribution of some densely distributed stations in the central area of Shanghai. This map was generated by ArcMap 10.8 (https://www.esri.com/en-us/arcgis/products/arcgis-desktop/overview).

Shanghai, located in East China, is a metropolis. According to the Shanghai Comprehensive Transport Operation Annual Report 2015, 15 metro lines (Lines 1–13, Line 16) and 339 metro stations were operated. The metro system is the most essential component of the transit system in Shanghai, and the average daily passenger traffic volume on the metro system accounts for approximately 43% of the total urban passenger traffic volume. Figure 1 shows the metro stations in Shanghai, including the spatial distribution of all metro stations and the details of some densely distributed stations in the central area. In studies related to metro station areas, the extent of the station area is usually defined as a buffer zone with a distance of 500–1500 m^7,18,20,36^, or it is defined as the range of how long people are willing to walk, such as 10 min, 15 min, etc.^8,9,37^.

The Master Plan of Shanghai attempts to establish a 15 min walkable neighborhood for residents, where residents can access all basic public services by walking. This study delineates a 15-min walking distance from the station as the metro station area in Shanghai^37^. We acquired an optimal walking path plan and time cost based on real-time road conditions by using the Amap web service, where the rail transit station was used as the origin, and we obtained the reachable time from each station. Finally, considering data availability, we derived a 15-min accessible area of 286 metro stations as our study area. In addition, there are overlapping and intersecting areas between adjacent stations. Considering that the intersecting parts are areas that are reachable by all related stations, we do not divide and allocate these intersecting parts so that they serve as the surrounding areas of all related stations.

### Data and preprocessing

#### POI data

The POI dataset used in this study comes from the Gaode Map platform (https://ditu.amap.com/). Gaode Maps are famous domestic providers of digital map content, navigation, and location service solutions. It provides POI data with 16 top-categories, 155 mid-categories, and 869 subcategories. First, we remove abnormal POI records with incomplete information and POI categories that are not closely related to functional characteristics, such as *Place Name & Address*, *Indoor Facilities*, *Pass Facilities*, etc. Then, the POIs within the station areas were extracted, including a total of 403,459 POIs. To satisfy the specific needs of analyzing the station area function, we reclasse the POIs into 11 categories from the perspective of travel purposes^26^, as shown in Table 1.

**Table 1:** POI category reclassification.

Top-categories	Mid Category in Gaode Map
Governmental Organization & Social Group	Governmental Organization, Foreign Organization, Democratic Party, Social Group, Public Security Organization, Traffic Vehicle Management, Industrial and Commercial Taxation Institution, etc
Recreation	Sports Stadium, Golf Related, Recreation Center, Holiday & Nursing Resort, Recreation Place, Theatre & Cinema
Residential	Commercial House Related, Residential Area
Food & Beverages	Chinese Food Restaurant, Foreign Food Restaurant, Fast Food Restaurant, Coffee House, Bakery, Dessert House, etc
Daily Life Service	Travel Agency, Ticket Office, Post Office, Logistics Service, Telecom Office, Beauty and Hairdressing Store, Repair Store, Photo Finishing, Lottery Store, etc
Shopping	Shopping Plaza, Convenience Store, Comprehensive Market, Stationary Store, Commercial Street, Clothing Store, etc
Medical Service	Hospital, Special Hospital, Clinic, Emergency Center, Disease Prevention Institution, Pharmacy, Veterinary Hospital
Working	Industrial Park, Building, Residential Area, Enterprises, Famous Enterprise, Company, Factory, Farming, Insurance Company, Securities Company, Finance Company, etc
Science/Culture & Education	Library, Science & Technology Museum, Planetarium, Cultural Palace, School, Research Institution, School, etc
Transportation	Airport Related, Railway Station, Coach Station, Commuter Bus Station, Taxi, Ferry Station, Ropeway Station, etc
Tourist Attraction	Tourist Attraction Related, Park & Square, Park & Plaza, Scenery Spot

#### Smart card transaction data

This study used transaction records data of Shanghai metro cards from 18 April 2015 to 24 April 2015; this week is a fairly regular week without holidays, which can reflect the general characteristics of station ridership. Each record of this dataset provides the card ID, travel date, travel time, station name, travel type, and deduction fee. We filter the original card records of bus trips. We match and combine two related smart card records into one metro travel record that includes information on the departure station, arrival station, departure time, arrival time, and trip time. We also remove erroneous records, including records with incomplete entries and exits, as well as records with travel times that are too short or too long^12^. Some examples of the complete travel records after processing are shown in Table 2.

**Table 2:** Examples of Shanghai metro card travel records after processing.

Card ID	Travel Date	Departure Time	Arrival Time	Departure Station	Arrival Station	Trip Time
5690	2015/4/20	8:23:50	8:40:51	Xingzhong Road	Yishan Road	17.017
5690	2015/4/20	18:05:16	18:20:07	Yishan Road	Xingzhong Road	14.85
5951	2015/4/20	17:54:45	18:10:25	Xincun Road	Jing’an Temple	15.667
…	…	…	…	…	…	…

## Methodology

**Fig. 2:**
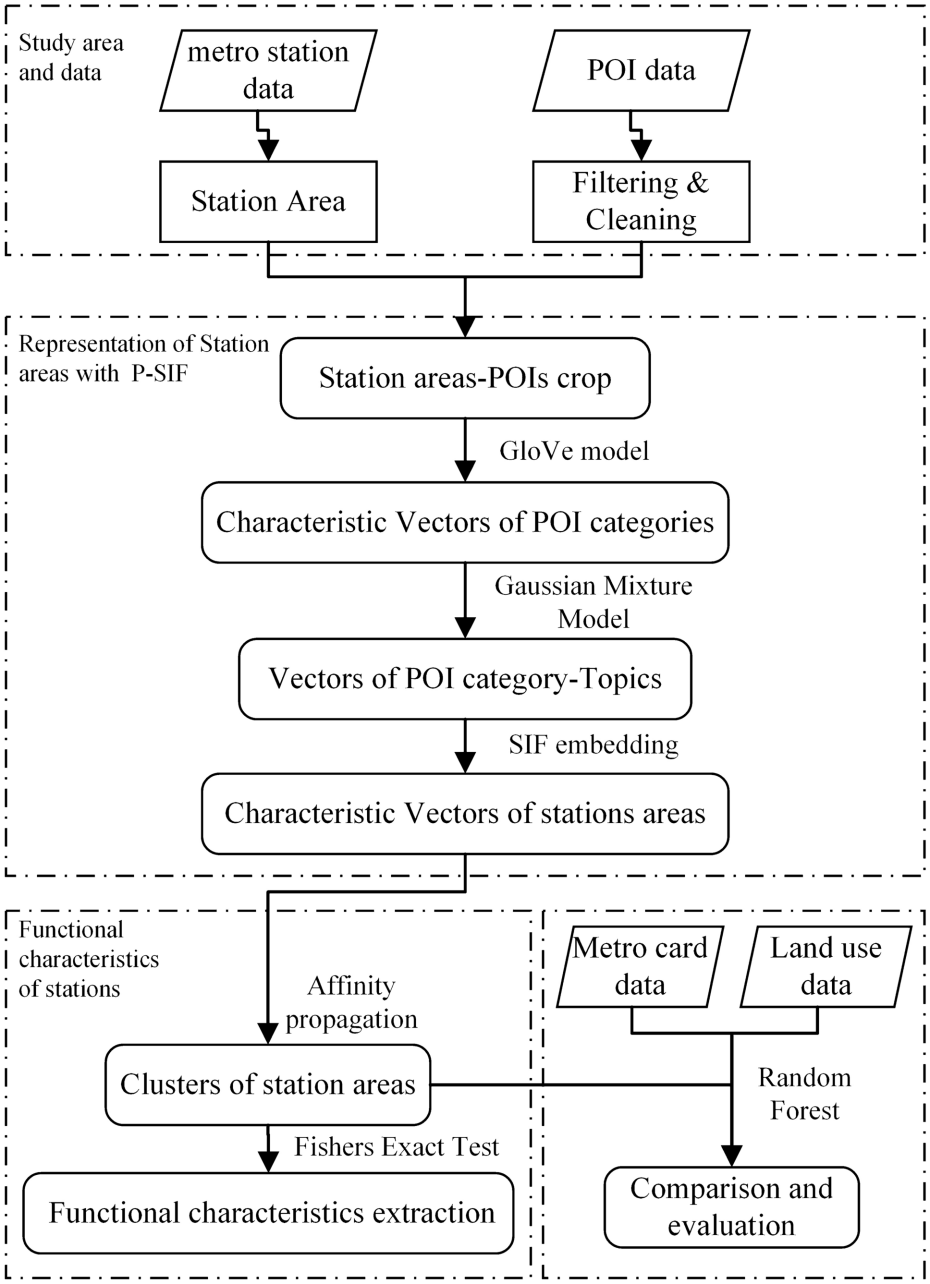
Flowchart of the extraction of metro station functional characteristics.

The flowchart of the proposed framework is illustrated in Fig. 2. The procedure includes four parts: (1) On the basis of the spatial co-occurrence information between POIs, we use the GloVe model to train the vectors of all POI categories. (2) By applying the P-SIF embedding method, we aggregate POI vectors within areas and compute the vector of each station area. (3) We cluster the station areas on the basis of the semantic vectors and annotate the functional characteristics of each area cluster. (4) The random forest algorithm is used to evaluate and compare the effectiveness of direct aggregation, TF-IDF weighted aggregation, SIF, and P-SIF embedding methods through metro passenger flow data and land use data.

### Training vectors of POI categories

The GloVe model is a word vector representation learning method that combines the advantages of two major model families: global matrix factorization and local context window methods^38^. GloVe utilizes the global statistical information of co-occurrence frequency in the corpus while simultaneously capturing meaningful semantic information via local context window methods. The co-occurrence patterns of various categories of POIs are highly important for understanding the semantics of the POIs in urban spaces. In addition, the GloVe model outperforms related models on similarity tasks and named entity recognition. Therefore, in this study, we use the GloVe model to train POI category vectors. Glove first constructs the co-occurrence matrix of words according to the corpus, and sets a weighted least-square regression model as the loss function. The parameters are calculated by the word co-occurrence probability in the window, and the parameters are updated continuously in the sliding window. Finally, the optimal parameter model is obtained, and the word vector is obtained according to the model. In this article, corpus refers to the POI in all the station areas, windows refer to each station area, and words refer to each POI type. The training process of the GloVe model is as follow.

First, we construct the co-occurrence matrix on the basis of the corpus. Because the POIs in station areas have no sequential order, we cite the greedy algorithm-based shortest-path model proposed by Yao^30^ to organize the sequential order of the POIs in the area. This method converts spatial location association relations of POIs to contextual relations of words in the document. Each station area document based on the POI shortest path sequence is obtained, and a station area corpus based on the shortest path order of station area documents is constructed. The POI co-occurrence matrix $X$ based on the contextual window is constructed from the station area corpus, where ${X}_{i,j}$ indicates the number of occurrences of POI category $j$ in the context of POI category $i$. On the basis of the distance $d$ of the two POIs in the context window, the weights are calculated via the decreasing weighting function:$decay=1/d$, so that the more distant the POI pair is, the smaller the weight of ${X}_{i,j}$.

The co-occurrence matrix $X$ is subsequently used as the input to the GloVe model for training. The goal of the GloVe model is to ensure that the final trained POI vector contains the information contained in the co-occurrence matrix. The basic principle is to construct an approximate relationship between hypothesis function $F$ of the POI category vectors (${v}_{i},{v}_{j},{v}_{k}$) and the probability information of the contribution matrix. By minimizing the value of the loss function $J$ between them, the final POI category vectors and the corresponding model are trained. The derivation process is as follows:

We define ${P}_{i,j}$ as the probability that POI category $j$ appears in the context of POI category $i$:

1 $${P}_{i,j}=P\left(j|i\right)=\frac{{X}_{i,j}}{{X}_{i}}$$

where ${X}_{i}$ is the number of times any POI categories are in the context of POI category $i$. According to existing research^39^, compared with probability of each POI category, the ratio is better able to distinguish relevant POI categories (shopping center and cinema) from irrelevant POI categories (hospital and cinema), and it is also better able to discriminate between two relevant POI categories. Therefore, we let the ratios of co-occurrence probabilities $\frac{{P}_{i,k}}{{P}_{j,k}}$ be the co-occurrence information for word vector learning rather than the probabilities themselves. Therefore, the model can be expressed in the following form:

2 $$F\left({v}_{i},{v}_{j},{v}_{k}\right)=\frac{{P}_{i,k}}{{P}_{j,k}}$$

$F\left({v}_{i},{v}_{j},{v}_{k}\right)$ is the POI vector function. $\frac{{P}_{i,k}}{{P}_{j,k}}$ is the the ratios of co-occurrence probabilities. Where ${v}_{i}$, ${v}_{j}$, and ${v}_{k}$ denote the POI category vectors $i$, $j$, and $k$, respectively. Since vector spaces are inherently linear structures and the ratio is a scalar, the difference and dot product on the two POI vectors are used in the construction of the function $F$. The following equation is derived:

3 $$F\left({v}_{i},{v}_{j},{v}_{k}\right)=F\left({\left({v}_{i}-{v}_{j}\right)}^{T}{v}_{k}\right)$$

Considering the symmetry of the symbiotic matrix, The following formula can be obtained:

4 $$F\left({\left({v}_{i}-{v}_{j}\right)}^{T}{v}_{k}\right)=\frac{F\left({v}_{i}^{T}{v}_{k}\right)}{F\left({v}_{j}^{T}{v}_{k}\right)}$$

Combining Eq. (2). The equation is simplified to:

5 $$F\left({v}_{i}^{T}{v}_{k}\right)={P}_{i,k}=\frac{{X}_{i,k}}{{X}_{i}}$$

Same as formula 5, $F\left({v}_{j}^{T}{v}_{k}\right)={P}_{j,k}=\frac{{X}_{j,k}}{{X}_{j}}$ and $F\left({v}_{i}^{T}{v}_{j}\right)={P}_{i,j}=\frac{{X}_{i,j}}{{X}_{j}}$. To make a linear fit to the formula, let $F=exp$, and add bias, $F\left({v}_{i}^{T}{v}_{j}\right)=\frac{{X}_{i,j}}{{X}_{j}}$ convert to Eq. (6) as:

6 $${v}_{i}^{T}{v}_{j}+{b}_{i}+{b}_{j}=\text{log}\left({X}_{i,j}\right)$$

where ${b}_{i}$ and ${b}_{j}$ are biases. The loss function is constructed by the least square regression model. According to the principle that the higher the frequency of co-occurrence is, the greater the weight of the POI category, a weight term is added to obtain the loss function of the model as:

7 $$J=\sum_{i,j}^{N}f\left({X}_{i,j}\right){\left({v}_{i}^{T}{v}_{j}+{b}_{i}+{b}_{j}-\text{log}\left({X}_{i,j}\right)\right)}^{2}$$

where $f\left({X}_{i,j}\right)$ is the weight function. To satisfy the conditions that $f(x)$ should be nondecreasing, that $f(x)$ has an upper bound and that $f(0) = 0$, the weight function is as follows:

8 $$f\left(x\right)=\left\{\begin{array}{c}{\left(\frac{x}{{x}_{max}}\right)}^{0.75}, x<{x}_{max}\\ 1, x\ge {x}_{max}\end{array}\right.$$

In NLP tasks, the vector dimension $dim$ is usually set between 50 and 1000. Considering that the number of POI category vectors in our experiment is much less than the number of words^5^, we set the POI category vector dimension to 100. In addition, to analyze the relationships among POI category vectors more intuitively, we map the high-dimensional POI category vector into a two-dimensional semantic space. Uniform Manifold Approximation and Projection (UMAP) and t-distributed Stochastic Neighborhood Embedding (t-SNE) are both commonly used dimensionality reduction algorithms, but they have some differences in their algorithms. tSNE uses the KL divergence as the loss function, which penalizes vectors that are close in low dimensions and far in high dimensions less heavily. However, UMAP uses the binary cross-entropy as a loss function, which penalizes both low-dimensional close and high-dimensional far or low-dimensional far and high-dimensional close more heavily, so UMAP is better able to reflect the true global structure. In this article, the GloVe model was trained to consider the correlation between POI types, so using UMAP for dimensionality reduction can better preserve the correlated features of POI category^40,41^.

### Computing characteristic vectors of station areas

Supported by the GloVe model, we obtain the characteristic vectors of all POI categories. Then, we need to adopt some method to obtain the station area vectors by aggregating the POI category vectors. We adopted the P-SIF model. P-SIF is a Document Embedding method used to represent long documents^35^. The main idea is to add word subject information for document aggregation; words within the same topic partition are weighted and averaged by the proportional weights of the topics to which they belong, and words in different topic partitions are concatenated $(\oplus )$ so that words belonging to different semantic topics are separated by concatenation $(\oplus )$, while words of similar topics are simply averaged. The final document vector ${v}_{{d}_{n}}$ is a more accurate semantic representation in a higher $n \times d$ dimensional vector space (*n* is the number of topics, and *d* is the dimensionality of the word vector). P-SIF retains the simplicity of simple weighted word averaging while taking into account the topical structure of the document and the importance of the words.

The P-SIF method is applied to aggregate POI category vectors into station area vectors, and the main steps are as follows:

(1) Topic classification for POI category Vectors.

This research uses a clustering algorithm to classify POI categories with similar semantic embedding vectors into the same topic. However, the same category of POI in different regional spaces can indicate different functional topics; for example, a restaurant may belong to the theme of a shopping mall or may belong to the theme of a scenic spot. Therefore, we use the Gaussian mixture model (GMM) for clustering. The GMM model is an overlapping clustering algorithm that allows the POI to belong to multiple topics and can be targeted to solve the problem.

In the GMM model, the number of topics $K$ is determined on the basis of the Akaike information criterion (AIC) and Bayesian information criterion (BIC) metrics. AIC is a measure of the complexity and fitness of the estimated model on the basis of information entropy. The BIC considers the sample size on the basis of the AIC to prevent excessive model complexity caused by high-precision models when the sample size is too large. In theory, the smaller the values of the AIC and BIC are, the better the model is.

The GMM assumes that each POI category is represented by a mixture model with K single Gaussian distributions, and the probability density function of the POI generated by the Gaussian mixture model is Eq. (9):

9 $$P\left({v}_{i}\right)=\sum_{k=j}^{K}p\left(k\right)p\left({v}_{i}|k\right)=\sum_{k=j}^{K}{\pi }_{k}N\left({v}_{i}|{\mu }_{k},\sum k\right)$$

where ${v}_{i}$ is the POI category vector; $K$ is the number of Gaussian mixture models, which also denotes the number of topics; $p\left(k\right)={\pi }_{k}$ is the weight of the $k$ th Gaussian model, that is, the probability of selecting the $k$ th model, satisfying $\sum_{k=1}^{K}{\pi }_{k}=1$; and $N(v|{\mu }_{k},\sum k)$ is the probability density function of the $k$ th single Gaussian distribution, and the specific expression is Eq. (10):

10 $$N\left(v|{\mu }_{k},\sum k\right)=\frac{1}{\sqrt{2{\pi }_{k}\left|\sum k\right|}}{e}^{-\frac{1}{2}{\left({v}_{i}-{\mu }_{k}\right)}^{T}{\sum k}^{-1}\left({v}_{i}-{\mu }_{k}\right)}$$

Each POI category vector ${v}_{i}$ has a certain probability $p({v}_{i},k)$ of belonging to each cluster ${c}_{k}:$

11 $$p\left( {v_{i} ,k} \right) = \frac{{\pi_{k} N\left( {v_{i} {|}\mu_{k} ,\sum k} \right)}}{{\mathop \sum \nolimits_{j = 1}^{K} \pi_{j} N\left( {v_{i} {|}\mu_{j} ,\sum j} \right)}}$$

where ${\mu }_{k},\sum k$ are the parameters of the $k$ th single Gaussian distribution, the parameters of every single Gaussian distribution are estimated via maximum likelihood estimation, and the iterations are repeated until the value of the likelihood function converges:

12 $$\mu_{k} = \frac{1}{{N_{k} }}\mathop \sum \limits_{i = 1}^{N} p\left( {v_{i} ,k} \right)v_{i}$$

13 $$\sum k = \frac{1}{{N_{k} }}\mathop \sum \limits_{i = 1}^{N} p\left( {v_{i} ,k} \right)\left( {v_{i} - \mu_{k} } \right)\left( {v_{i} - \mu_{k} } \right)^{T}$$

where $${N}_{k}=\sum_{i=1}^{N}p({v}_{i},k)$$, and $${\pi }_{k}=\frac{{N}_{k}}{N}$$.

(2) POI-Topic Vector Formation.

For each POI category vector ${v}_{i}$, create $K$ different POI-cluster vectors of $d$ dimensions $c{v}_{ik}$ by weighting the POI category embedding with its probability distribution coefficient $p({v}_{i},k)$ in the $k$ th topic. All the $K$ POI-cluster vectors are then concatenated into an $K\times d$ dimensional embedding and weighted with an inverse document frequency (IDF) of ${v}_{i}$ to form a POI-topic vector $t{v}_{i}$.

(3) SIF Weight Averaging and Common Component Removal.

Smooth inverse frequency (SIF) is a sentence embedding method proposed by Sanjeev Arora (Arora et al. 2017). The main idea is to remove the common component in documents, i.e., to remove the noise and redundancy of the document vector and keep the relatively unique parts of each document, thus making the representation of the document more discriminating. Finally, for all POI appearing in the station area ${S}_{n}$, we weigh the POI-topics vectors $t{v}_{i}$ by the smooth inverse frequency $\left(\frac{a}{a+p(v)}\right)$. Next, we remove the common contexts from the station area by removing the first principal component from the weighted station area average vectors, and then, we obtain the station area vector ${v}_{{s}_{n}}$.

### Clustering the station area vectors

To classify all the station areas into different clusters on the basis of their semantic characteristics, we use the affinity propagation (AP) clustering method to cluster the vectors of the areas. The AP algorithm is a clustering approach that has been widely utilized in previous studies^9,17,23^. It is a graph theory-based clustering method^42^ that does not require determining the number of clustering centers in advance.

We use the Euclidean distance to define the similarity function $f(i,j)$ between station areas ${S}_{i}$ and ${S}_{j}$. The larger the value of $f(i,j)$ is, the closer the distance between ${S}_{i}$ and ${S}_{j}$, and the greater the ability of ${S}_{j}$ to be the clustering center of ${S}_{i}$ in the AP algorithm. The N*N similarity matrix is obtained on the basis of the similarity between the station area vectors. The diagonal elements are the phase distances between the samples themselves. These values are called the reference degree and theoretically have values of 0. Before the iteration begins, assume that all sample points have the same ability to become clustering centers, and the reference degree initialization is generally set to the median or minimum value of all values in the similarity matrix. A greater reference degree indicates that this data point is more capable of becoming a cluster center.

On the basis of the similarity function, the AP algorithm continuously updates the responsibility value *r* and availability value *a* of each point through:

14 $$r\left( {i,k} \right) \leftarrow f\left( {i,k} \right) - \max \left( {a\left( {i,k^{\prime}} \right) + f\left( {i,k^{\prime}} \right)} \right)$$

15 $$a\left(i,k\right)\leftarrow \text{min}\left(0,r\left(k,k\right)+\sum_{{i}{\prime}i,k}\text{max}(0,r({i}{\prime},k)\right)$$

16 $$a\left(k,k\right)\leftarrow \sum_{{i}{\prime}i,k}\text{max}(0,r({i}{\prime},k)$$

The responsibility value $r(i,k)$ assesses how suitable ${S}_{k}$ is to be regarded as the clustering center for ${S}_{i}$ compared with other candidate points for ${S}_{i}$. The availability value indicates how appropriate for ${S}_{i}$ to choose ${S}_{k}$ as its clustering center, considering other points’ preferences for ${S}_{k}$ as the clustering center. The responsibility value and the availability value of the sample points are summed to detect their decision to select a cluster center. The iteration is repeated until the cluster center no longer changes, the number of iterations exceeds a given number, or the decision about the sample points in a subregion remains unchanged; then, the algorithm ends.

### Extracting the functional characteristics of station area clusters

After classifying the station areas with similar semantic representations into clusters, we need to interpret these clusters. Owing to the highly unbalanced distribution of POI types, it is not sufficient to use only the number of POIs per type. Thus, we adopted a statistical significance testing approach to quantify the significance of a POI type to a station area. Fisher’s exact test (FET) is a statistical significance test proposed by Fishers for the analysis of 2*2 columnar tables. It works for small observations and calculates the exact probabilities rather than approximations. Relevant studies have been conducted to verify the rationality of applying FET to mine the functional characteristics of regions^43,44^.

In Table 3, variance 1 is a POI type, and variance 2 is a station area cluster. where $a$ is the number of one POI type in one cluster, $b$ is the number of other POI types in the same cluster, $c$ is the number of the same POI types in the other clusters, and $d$ is the number of other POI types in the other clusters.

**Table 3:** The 2 × 2 contingency table represents two different attributes used in Fisher’s exact test.

	**Variance 1**	**Non-Variance 1**
Variance 2	*a*	*b*
Non-Variance 2	*c*	*d*

The p value and odds ratio are two indicators that are calculated via FET. The formula for calculating the p value is as follows:

17 $$p-value=\frac{\left(a+c\right)!\left(a+b\right)!\left(b+d\right)!\left(c+d\right)!}{a!b!c!d!\left(a+b+c+d\right)!}$$

The p value quantifies the probability of observing that amount of POI type t in area cluster z by chance. The original assumption of FET is that the POI type and the area cluster in the columnar tables are independent of each other. The smaller the p value is, the more the original hypothesis is rejected, indicating that the POI type is more relevant to the area cluster and more important relative to that area cluster.

The formula for the odds ratio is as follows:

18 $$odd-ratio=\frac{a\times d}{b\times c}$$

The odds ratio can quantitatively describe the magnitude of the correlation, allowing us to visually compare the degree of correlation between different POI types and station area clusters. Thus, the p value and odds ratio are used to analyze the functional characteristics of the station area clusters.

## Results

### Embedding vectors and topic analysis of POI categories

#### Embedding vectors of POI categoryies

**Fig. 3:**
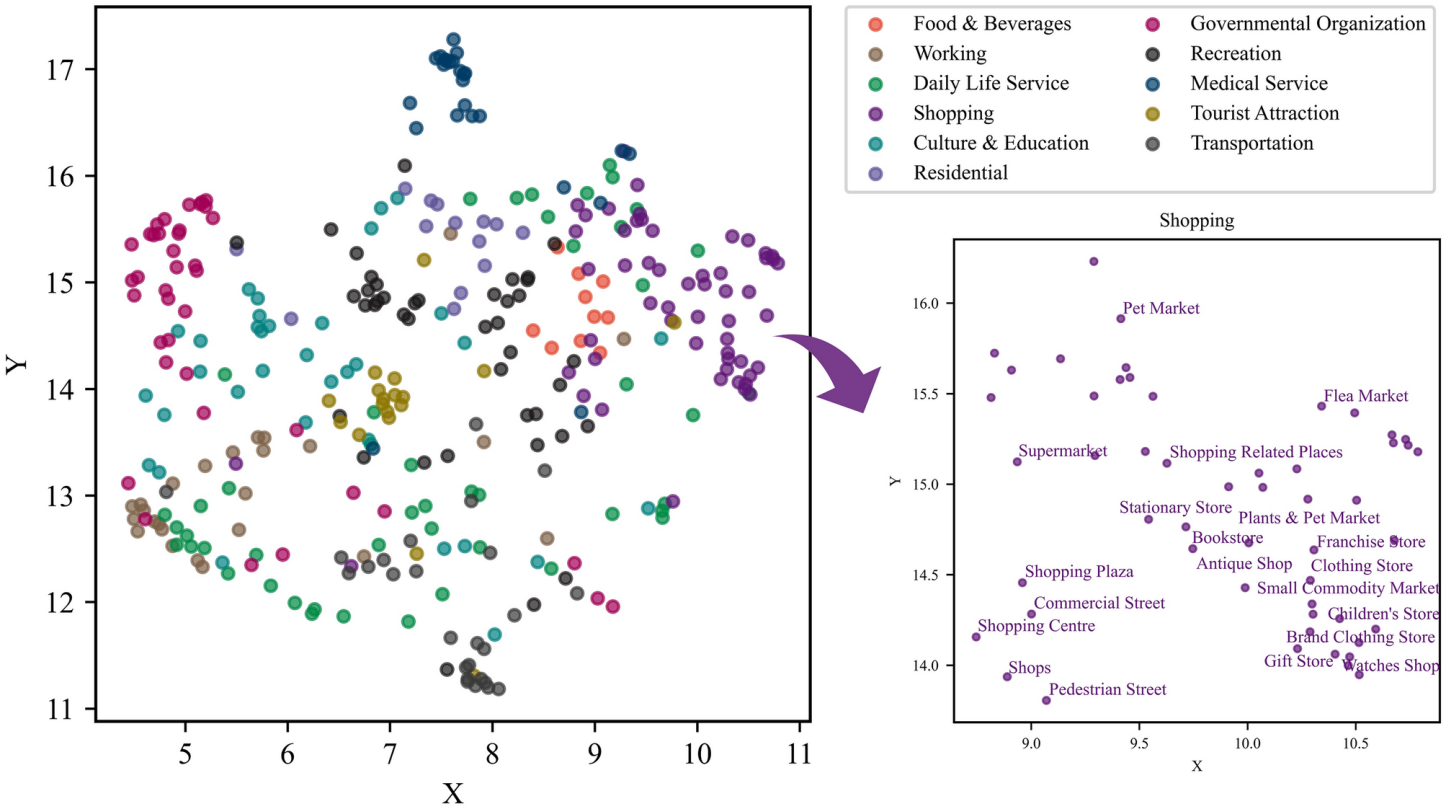
POI categories in semantic space. POI categories semantic space with the POI categories being reclassified according to the top-categories in the Gaode Map.

In the training of the GloVe model, to reflect the rich diversity of words in the corpus, we choose the subcategories of the POIs to organize the corpus. Additionally, to be consistent with the description of most POI types, we choose the mid-category category if the sub-POI category is a specific name; for example, the *subcategory KFCs* and *McDonald’s* categories are labeled mid-category *fast food restaurants*. Finally, we trained a total of 349 POI category vectors. Then, via the UMAP dimensionality reduction method introduced in Sect.**“Training vectors of POI categories”**, we map all POI category embeddings to a two-dimensional semantic space. To verify the semantic correctness of the POI vectors we have trained, we display them according to the reclassification type of the Gaode map. As shown in Fig. 3, POI categories belonging to the same types are presented in the same color. As it is difficult to display the names of all POI categories, Fig. 3 also shows the names of the POI categories belonging to the *shopping* type. This indicates that similar or related POI categories are close in semantic space, which is coincident with people’s cognition and verifies that the semantic features and relations of POI categories are well captured.

#### Topic analysis of POI categories

**Fig. 4:**
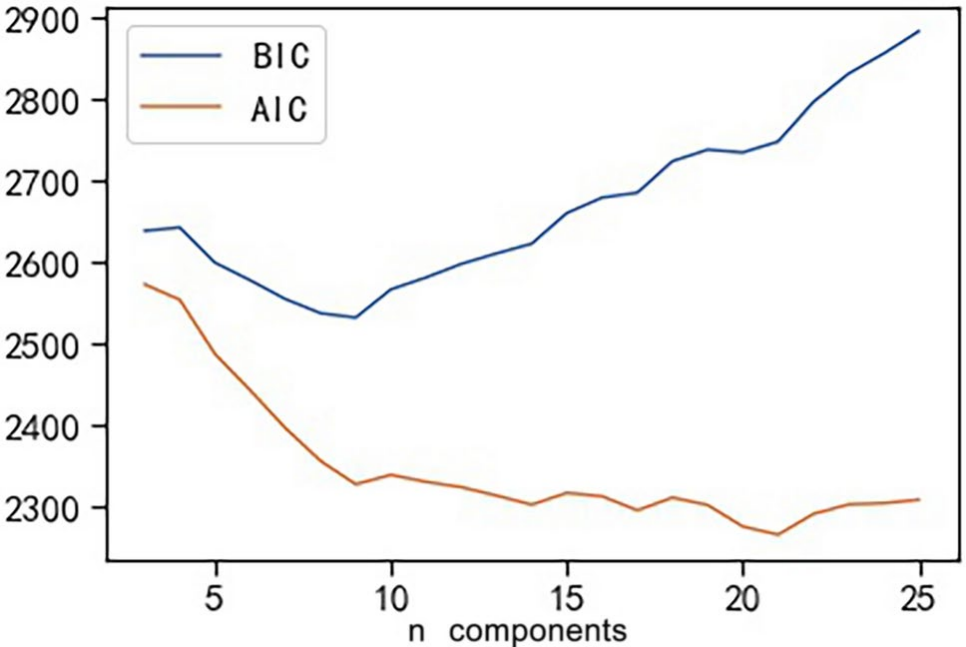
Metrics of the AIC value and BIC value for the GMM model.

We observed that the 11 POI types are not completely separate from each other and that the POI categories belonging to different types may also be close to each other in the semantic space in Fig. 3. Therefore, in this study, we extracted the topic information of the POI categories on the basis of their semantic features. We used the GMM model of Sect.**“Computing characteristic vectors of station areas”** to cluster the 349 POI category vectors into several corresponding semantic topics. To determine the topic number K, we adopted the AIC and BIC evaluation metrics introduced in Sect.**“Computing characteristic vectors of station areas”** for the GMM algorithm. As shown in Fig. 4, we set the K values from 3–26 and find that the AIC and BIC values are lower when K = 17; we choose K = 17 as the ideal number of clusters.

**Fig. 5:**
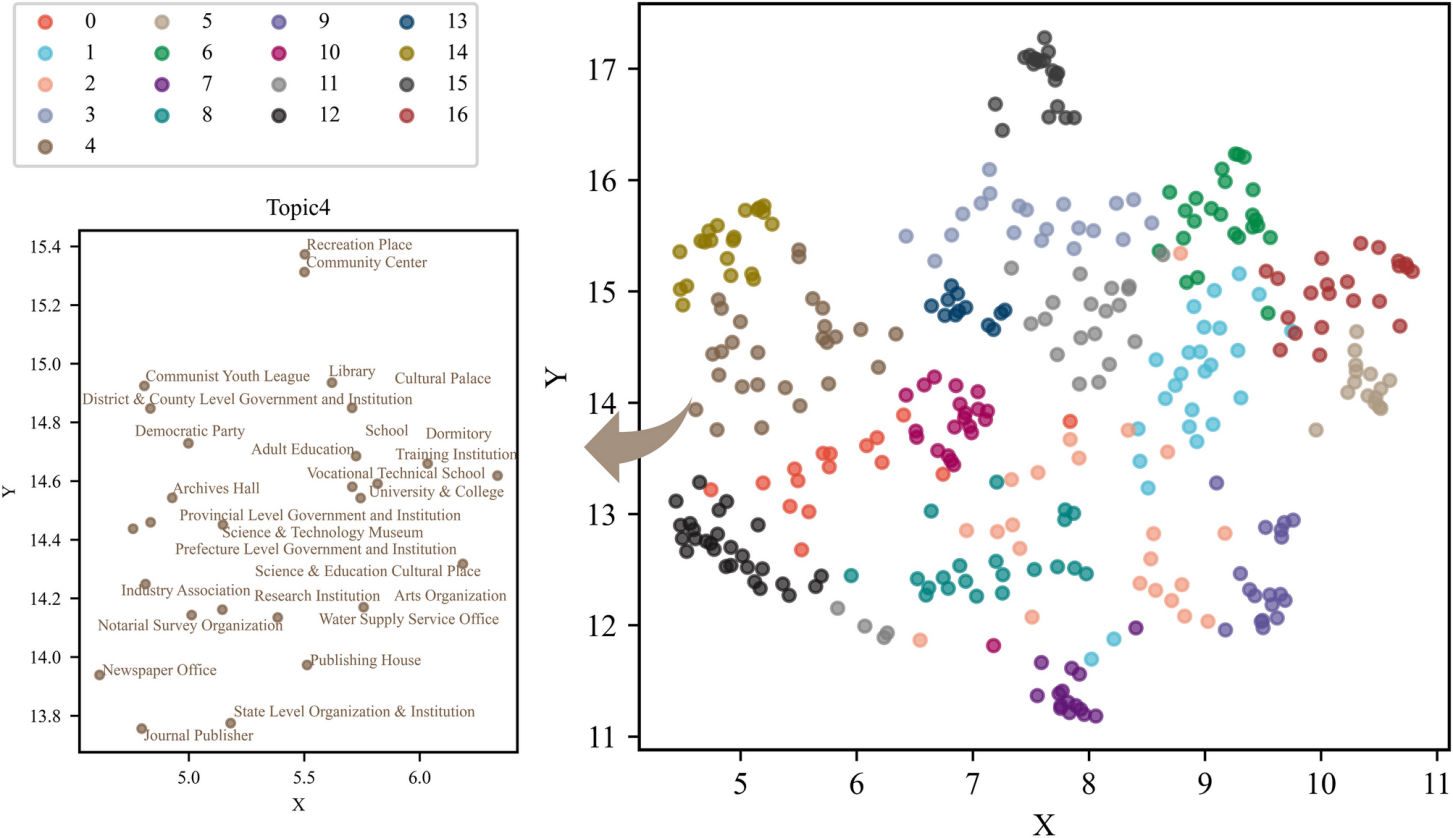
Topics in semantic space. POI categories semantic space with the types being classified according to the topics from GMM clustering.

To demonstrate the results of the topic features of the POI categories, we also displayed all the POI categories with topic information in two-dimensional semantic space through the UMAP dimensionality reduction method, as shown in Fig. 5. It shows the component POI categories of Topic 4 in detail. We can see that the results of the topic extraction are relatively compatible with the original classification of Gaode Map, that is, people’s perceptions. However, there are some differences between these two perspectives. For example, *Shopping Plaza*, *Convenience Store*, and *Stationary Store*, which all belong to the same top type of *Shopping* in Gaode Map, are grouped into three topics in semantic-based clustering. *Chinese Food Restaurant*, *Convenience Stores*, and *Pharmacy*, which are classified as *Food & Beverages*, *Shopping*, and *Medical Service* in Gaode Map, are defined as the same type of *Daily Life Service* in the theme classification. This indicates that these semantically related POI categories that work together to provide daily life services to residents are usually found in communities.

**Fig. 6:**
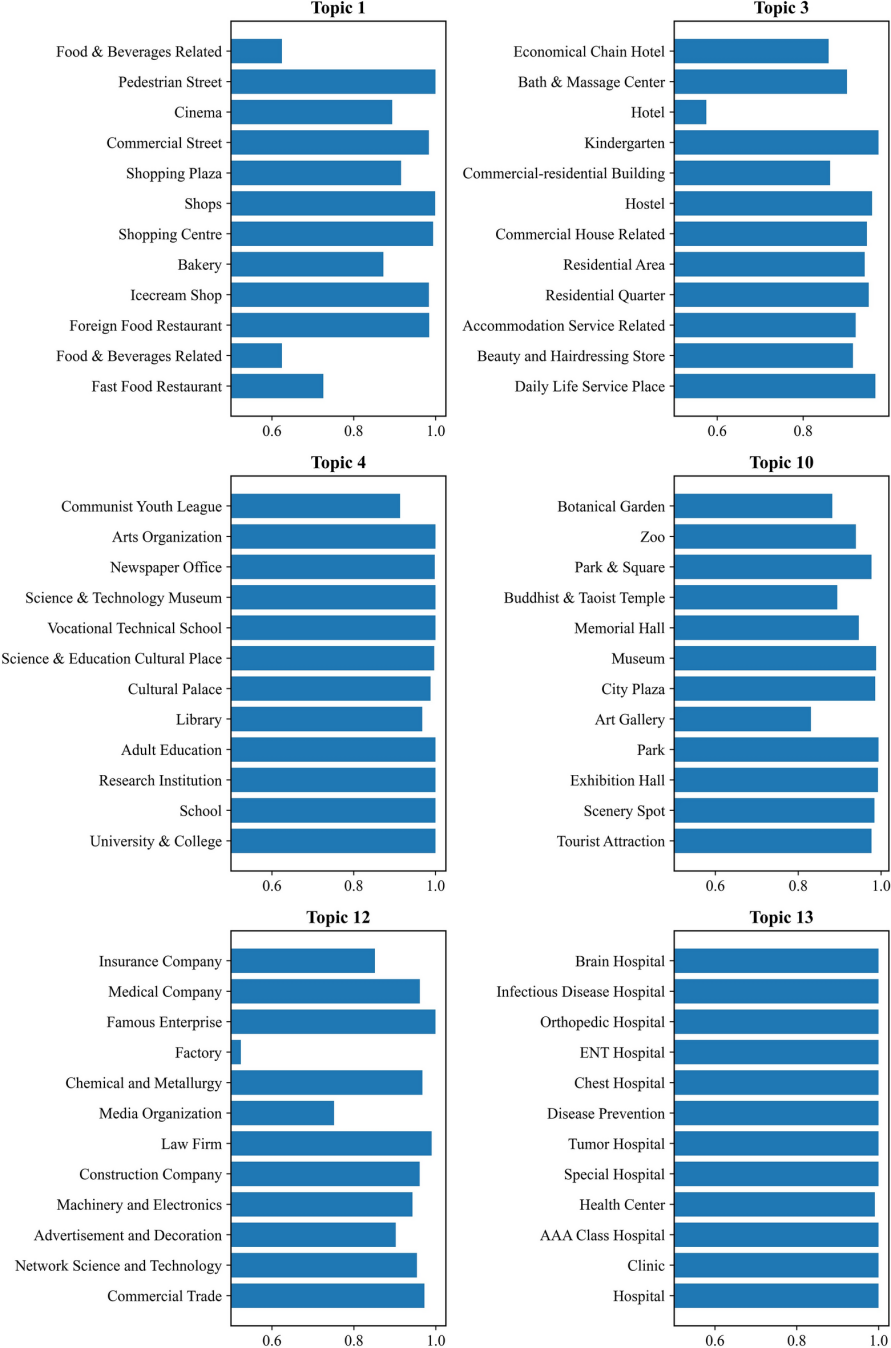
Six sample topics with their POI categories related to the POI categories semantic feature.

To specifically illustrate the topics extracted by the GMM models, we display several topics and their related POI categories with certain probabilities in Fig. 6. Specifically, Topic 1 is a shopping topic that includes *Shopping Centre*, *Shops*, *Shopping Plaza*, *Commercial Street*, and *Pedestrian Street* with high probabilities. Topic 3 represents a residential-related topic that contains *residential quarter, residential area, hotel, daily life service*, etc. Topic 4 is an educational topic that consists of various frequently occurring POI categories, including *research institution*, *University & College*, *School*, *Library*, and *Science & Technology Museum*. Topic 10 indicates a tourist-related topic that contains *the tourist attraction, scenery spot*, *Park & Square*, *City Plaza*, *Zoo,* and *Botanical Garden*. Topic 12 represents a working-related topic that consists of various business companies, such as *Network Science and Technology*, *Advertisement and Decoration*, *Machinery and Electronics*, *Insurance Company*, and *Medical Company*. Topic 15 is a healthcare topic that includes hospitals, clinics, health centers, and special hospitals. In addition, to demonstrate the polysemantic features of some POI categories, Table 4 shows several example POI categories and their probabilities of being classified into different topics by the GMM model. For example, *Coffee House*, which is a typical polysemous POI category in urban areas, exhibits significant correlations with topic 1 and topic 11, which represent *Shopping* and *Recreation, respectively*. The results indicate that the GMM model can discriminate and describe the polysemantic features of the same POI category belonging to different topics.

**Table 4:** POI categories with multiple senses are assigned to multiple clusters with significant probabilities.

POI Category	Topic	POI categories in Topic	Prob
**Chinese Food Restaurant**	1	Fast Food Restaurant, Foreign Food Restaurant, Bakery, Dessert House, Shopping Centre, Shops	0.648
	6	Convenience Store, Fruits Market, Supermarket, Comprehensive Market	0.226
**Coffee House**	1	Fast Food Restaurant, Foreign Food Restaurant, Bakery, Dessert House, Shopping Centre, Shops	0.376
	11	Pub, Tea House, Recreation Center, KTV, Beach	0.509
**Franchise Store**	5	Clothing Store, Children’s Store, Brand Shoes Store, Jewelry Store, Sports Store, Gift Store	0.566
	16	Shopping Related Places, Antique Shop, Flower Shop, Furniture Store Home, Electronics Hypermarket	0.433
**Hotel**	3	Residential Quarter, Accommodation Service Related, Commercial House Related, Residential Area	0.575
	11	Four-star Hotel, Five-star Hotel, Recreation Center, Pub	0.421
**Pharmacy**	3	Residential Quarter, Daily Life Service Place, Commercial House Related, Residential Area	0.385
	6	Convenience Store, Medical Supplies, Supermarket, Comprehensive Market	0.602

### Functional characteristics analysis of station area clusters

**Fig. 7:**
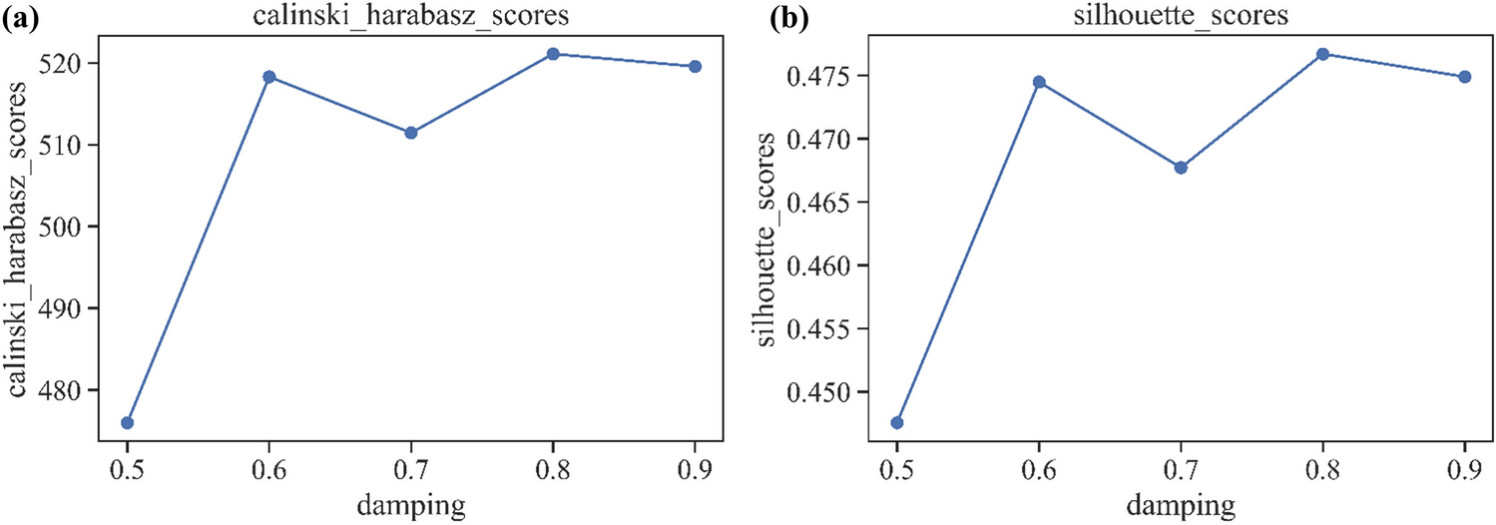
Evaluate the best damping value. (a) the Calinski–Harabasz score (CH) using different damping value in AP algorithm; (b) the silhouette score using different damping value in AP algorithm.

**Fig. 8:**
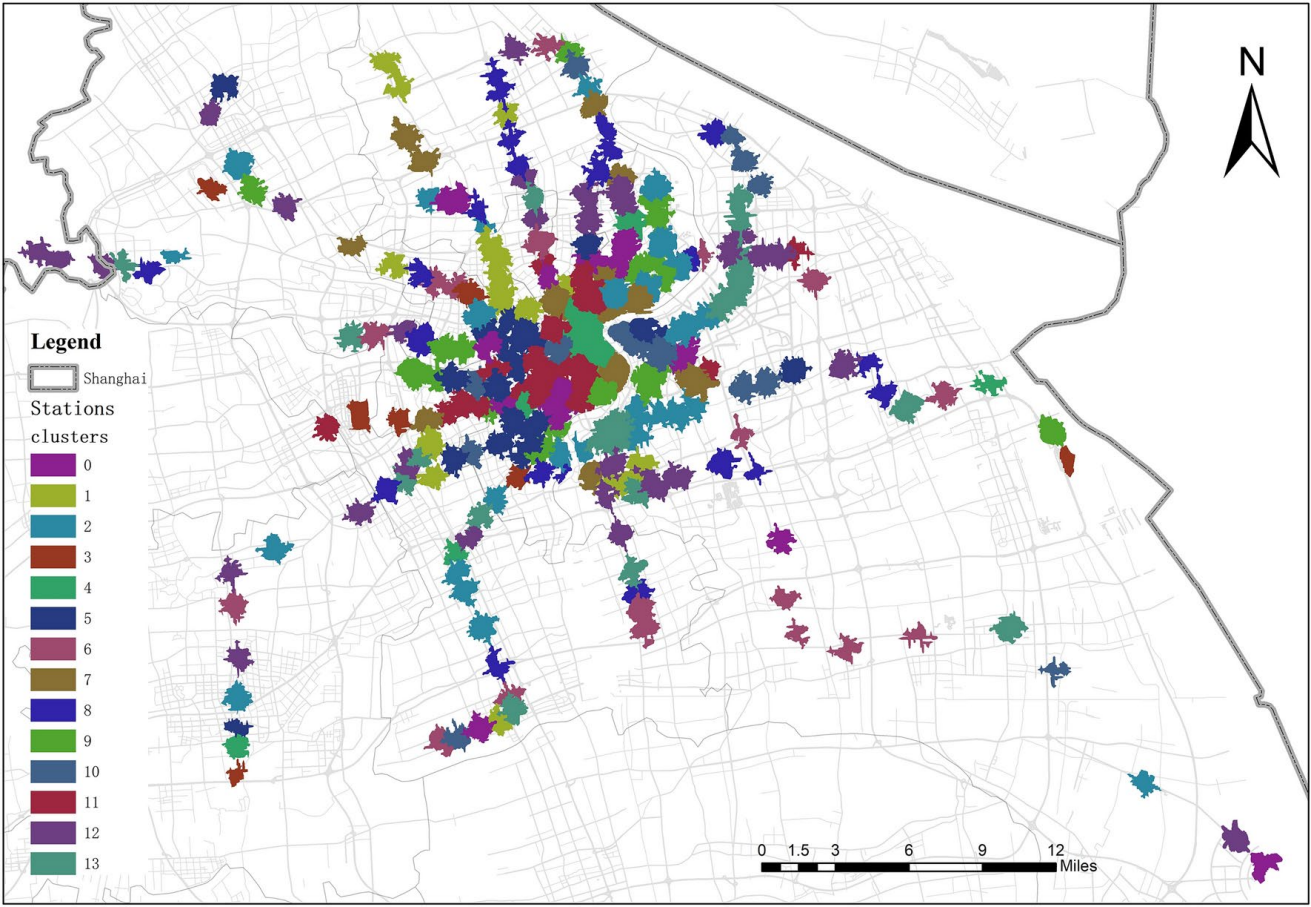
Clusters of metro station areas with their functional characteristics. This map was generated by ArcMap 10.8 (https://www.esri.com/en-us/arcgis/products/arcgis-desktop/overview).

The P-SIF model introduced in Sect.**“Computing characteristic vectors of station areas”** obtains the semantic feature vectors for 286 station areas by aggregating the POI categories within areas. To identify the functional types of station areas, we apply the AP algorithm to cluster the vectors of station areas and aggregate stations with similar semantic features into the same functional cluster. We determined the parameters of the AP algorithm on the basis of two metrics: the Calinski–Harabasz score (CH) and the silhouette score. Higher values of CH and silhouette scores indicate a better clustering effect. Figure 7 shows the CH scores and silhouette scores for parameters ranging from 0.5–1. As the value of the damping factor changes, the CH scores and silhouette scores tend to be stable. Therefore, we choose 0.6 as the optimal damping factor to cluster the vectors of station areas and divide station areas into 14 functional clusters. The spatial distributions of these station area clusters are shown in Fig. 8, where different colors indicate different functional clusters.

**Fig. 9:**
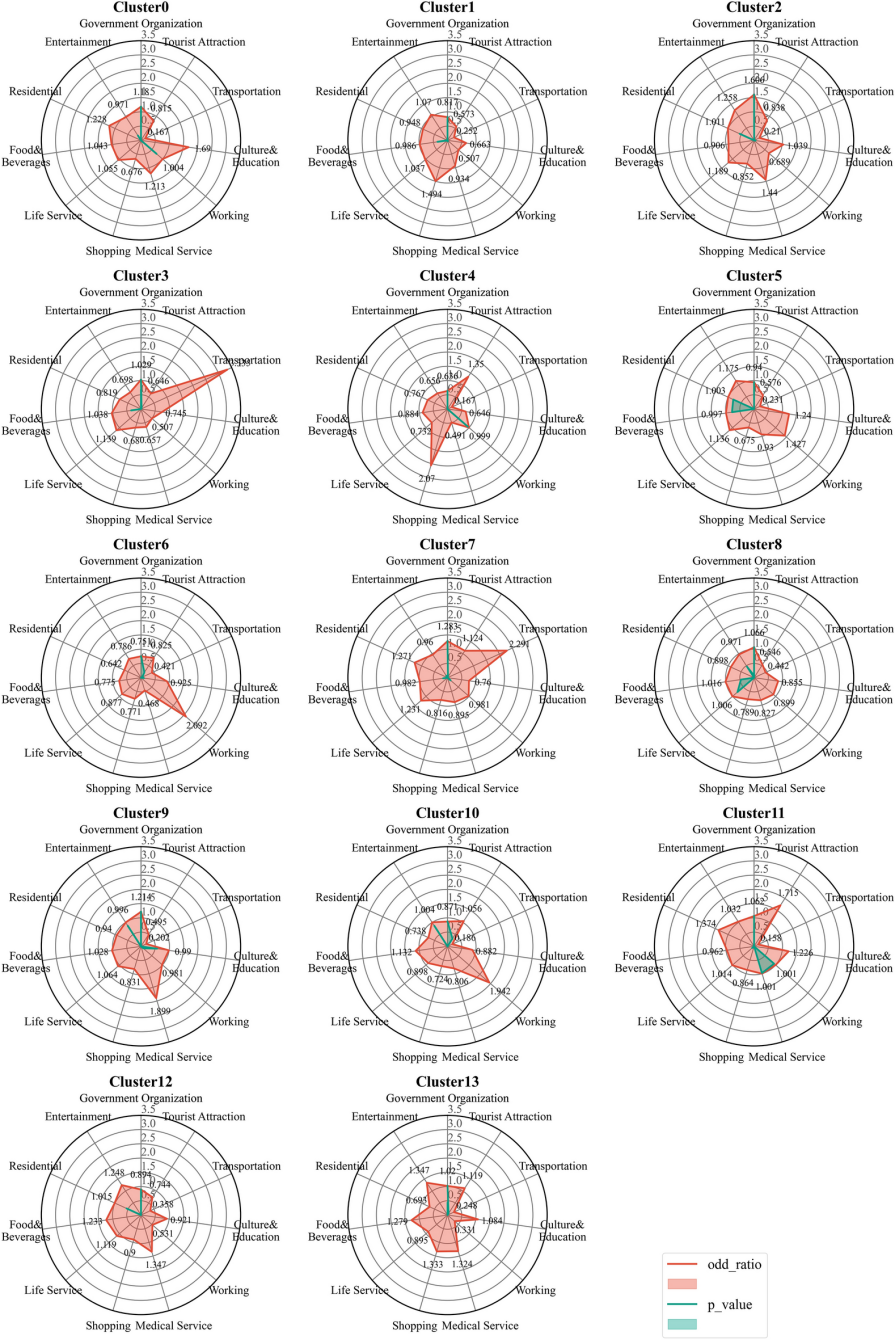
Odd-ratio score and p value of the POI types in each station area cluster.

**Fig. 10:**
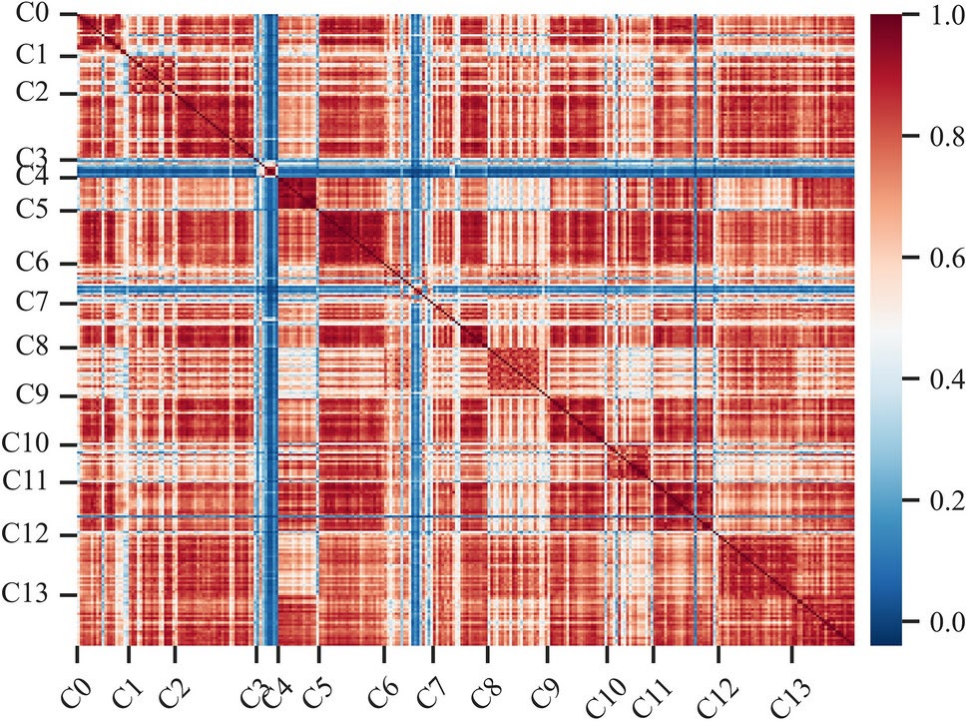
Heatmap of functional similarities between metro station clusters.

**Fig. 11:**
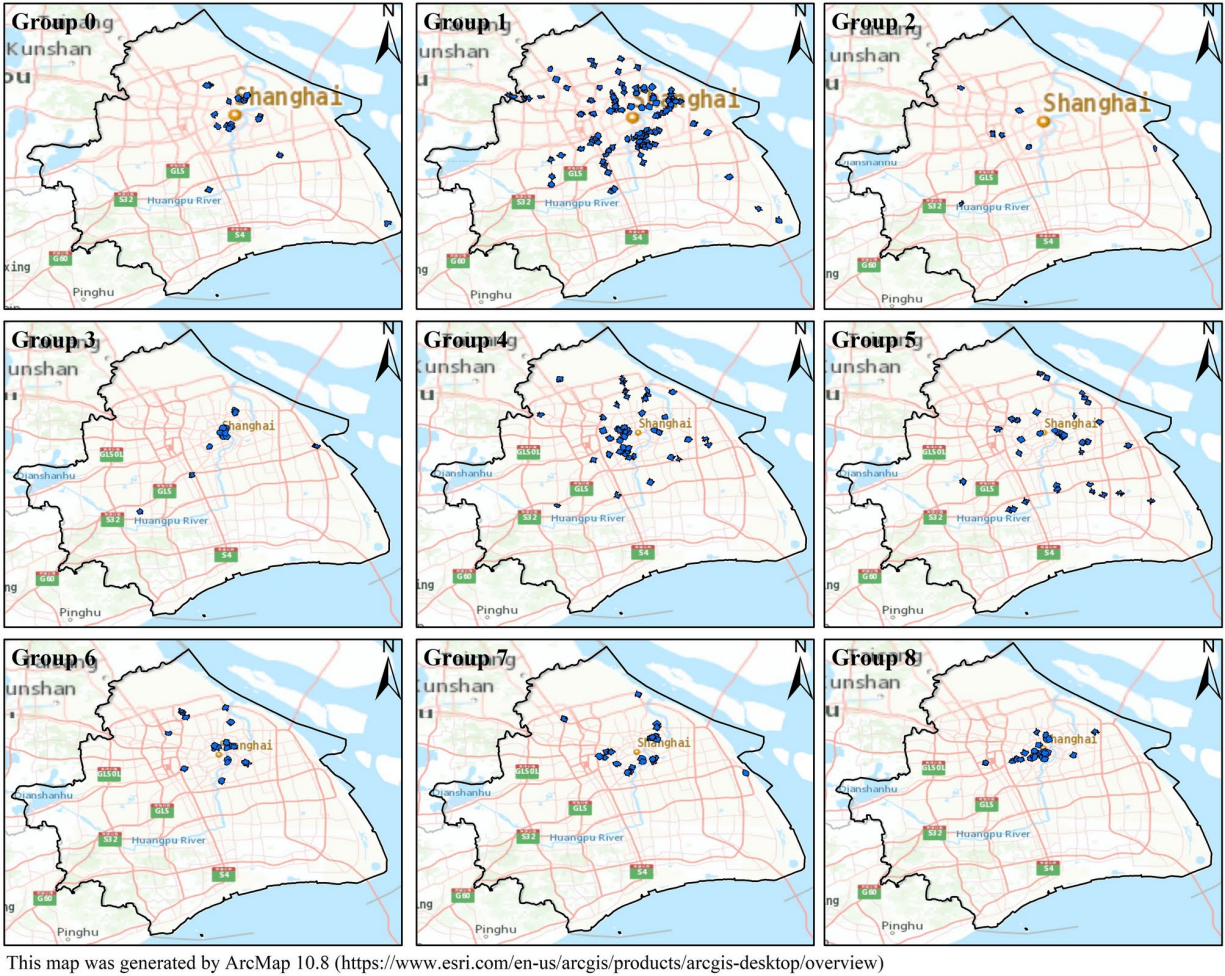
The spatial distribution of metro station areas in Groups 0 to 8. This map was generated by ArcMap 10.8 (https://www.esri.com/en-us/arcgis/products/arcgis-desktop/overview).

To understand the functional characteristics of the station area clusters, we used the FET explained in Sect.**“Extracting the functional characteristics of station area clusters”** to annotate these clusters. Figure 9 visualizes the odd-ratio and p value results of the FET for 14 station area clusters (C0–C13). We find that the POI configuration results for some station area clusters are relatively similar. Therefore, we used the cosine distance between the vectors of the station area to measure the functional similarity of the station area and further explore the similarities and differences between station area clusters. By calculating the cosine distance between all pairs of station area vectors, we produce a 286-by-286 similarity matrix. As shown in Fig. 10, each element in the matrix refers to the similarity value calculated by the cosine distance between the characteristic vectors of the station areas. It is colored according to the similarity value with a range from 0 to 1, where red elements indicate high similarity and blue elements indicate low similarity. The matrix is sequentially reindexed according to cluster codes from Cluster 0 to Cluster 13. The names and codes of the clusters are labelled on the axes. The results show that the station areas shared a functional similarity within their clusters, whereas the station areas in other clusters had lower similarity scores within their clusters. We also found that similarities also appear in cluster pairs between Clusters 1 and 2 and between Clusters 12 and 13. On the basis of the POI configuration characteristics and the similarity of the 14 clusters computed via FET and the cosine distance, we reclassified them into nine groups. Figure 11 shows the separate geographical distributions of Groups 0 to 8.

Group 0 (educational and residential mixed type): Based on the results of the FET, Cluster 0 in Fig. 10 shows a high odds ratio and low p values for the type of *science/culture and education*, representing the dominant position of the educational function in this cluster. The cluster mainly includes Shanghai University Station, Nanchen Road Station, Tongji University Station, Chifeng Road Station, Guoquan Road Station, Shanghai Science and Technology Museum Station, etc. According to the Gaode map, many educational and scientific institutions are located near these stations. Taking the Chifeng Road station as an example, the Shanghai Institute of Technical Physics of the Chinese Academy of Sciences, Shanghai International Studies University, and Shanghai University of Finance and Economics are located on the east, south, and west sides of the station, respectively. Similarly, scientific institutions and universities such as the Shanghai Institutes for Biological Sciences Fudan University, Shanghai Medical College of Fudan University, and ShanghaiTech University are distributed within the area of Zhaojiabang Road Station. In addition, the cluster has a more balanced configuration in other categories, such as residential, medical, work, and life services facilities, indicating that the residential type is another important function of the cluster. Therefore, we define the cluster as a mixed educational-residential type.

Group 1 (residential type): We merged Clusters 1, 2, 12, and 13 into this group and inferred that it is a residential function. Although the odd-ratio values of the highest type in these four clusters are not consistent, the odd-ratio values in the highest type do not differ significantly from those in the other types but remain relatively balanced. These clusters have basically the same configuration of facilities, including residential services, living services, restaurants, shopping, and medical services, reflecting a relatively mature residential and living configuration. Therefore, we interpret them as residential functions. The stations in this group include Jinyun Road Station, Jiuting Station, Sijing Station, Sheshan Station, Chuansha Station, and Tangzhen Station. These stations are located in typical residential areas such as JiangQiao Town of Jiading District, Sheshan Town and Sijing Town of Songjiang District, Chuansha town, and Tang town of Pudong New Area.

Group 2 (transportation type): In Cluster 3, the transportation type has a high odds ratio score, indicating that transportation services are a representative function in this cluster. The functional cluster contains several representative stations that are close to transportation institutions, e.g., Shanghai West Railway Station, Shanghai South Railway Station, Hongqiao Railway Station, Hongqiao Airport Terminal 1 Station, and Hongqiao Airport Terminal 1 Station.

Group 3 (commercial type): The p value and odds ratio results of Cluster 4 indicate that shopping and tourist attractions are representative facilities in these station areas. In terms of spatial distribution, most of these stations are located in typical commercial centers such as East Nanjing Road, North Sichuan Road, Yu Garden, Zikawei, and Wujiaochang. Among them, People’s Square Station and East Nanjing Road Station and its surrounding stations are clustered in bustling commercial areas of the central city. Other stations, such as Xinzhuang Station and Yuandong Avenue Station, are also surrounded by commercial centers, shopping malls, and city parks. As cities have grown, these places have developed into a new type of social place that combines business, recreation, and tourism.

Group 4 (working and residential mixed type): Clusters 5 and 8 have a similar configuration of facility categories as Group 0 does, which is a relative balance of each type. However, the difference is that the working type in the residential group has a relatively low value, whereas the office type in Clusters 5 and 8 presents equal strength with the residential and living services categories. Guilin Road Station, Jiangsu Road Station, West Yan’an Road Station, Luoshan Road Station, and other stations are included in this group. The surrounding built environments of these stations include various enterprise buildings and residential communities. Therefore, we interpret the group as a mixture of residential and working functions.

Group 5 (working type): The working types in Clusters 6 and 10 highlight a higher odd value and reflect the functional theme of working. Specifically, the Minhang Development Zone Station, Tieli Road Station, Jinhai Road Station, Shendu Highway Station, and Lianhang Road Station in Clusters 6 are located near the Minhang Economic and Technological Dev Industry Base, Baoshan Industry Base, Jinqiao Industrial Park, Pujiang High Tech Park, and Caohejing Development Zone, respectively. Clusters 10 includes Lujiazui Station, West Nanjing Road Station, North Waigaoqiao Free Trade Zone Station, Caohejing Hi-Tech Park Station, and other stations. Among them, places such as West Nanjing Road and Lujiazui, located in the inner ring, are traditional employment centers in Shanghai. Industrial parks such as Zhang Jiang high tech Park, the Waigaoqiao Free Trade Zone, and the Caohejing Economic and Technological Development Zone on the edge of the central city have many office buildings, such as technology buildings, industrial buildings, and trade buildings.

Group 6 (transportation and residential mixed type): The POI types of Cluster 7 have a balanced configuration, whereas the transportation type has a high odd-ratio value, reflecting the representation of transportation and residential functions. Specifically, the Songbin Road station area contains the Wusong Long-Distance Bus Terminal; the International Cruise Terminal Station, the surrounding Tilanqiao Station, and other stations are located on the west side of the Huangpu River, and its areas may involve the wharf on the river bank. Shanghai Railway Station and its neighboring North Zhongshan Road Station and Zhongxing Road Station can reach Shanghai Railway Station within the area of these three stations. According to the Gaode map, we analyse why Shanghai Railway Station is not divided into the previous transportation group, probably because a more complete living place has formed around it with a high density of mixed functions.

Group 7 (healthcare and residential mixed type): The FET results for Cluster 9 reflect the representation of medical and residential functions, with the cluster having a predominantly residential area within the station area, whereas hospitals play an important role in the cluster. The cluster contains Beixinjing Station, Zhenbei Road Station, Youyi Road Station, and Xiangyin Road Station. Many hospitals are located within these station areas. For example, the three stations of Shanghai Children’s Medical Center Station, Tangqiao Station, and Lancun Road Station are distributed around Renji Hospital and Shanghai Children’s Medical Center. The Youyi Road station area can reach the Shanghai Ninth People’s Hospital and the Hospital of Integrative Medicine.

Group 8 (tourism, working, and residential mixed type): Cluster 11 includes Shanghai Circus World Station, Zhongshan Park Station, Xintiandi Street Station, Laoximen Station, etc. The cluster has the highest odds ratio value for the type of tourist attraction, indicating the tourism function of the cluster, while residential, office, medical, and shopping also have a relatively balanced configuration, indicating that residential also plays an important role within the areas. Many tourist attractions and well-known places are located within the cluster area. For example, Hongkou Football Stadium Station is adjacent to Hongkou Football Stadium and Luxun’s Park, and the station area of Jing’an Temple contains famous places such as Jing’an Temple, Jing’an Park, and Bailemen Hotel. Zhong Park Station, which is close to Zhongshan Park, is a famous attraction. These stations are surrounded by a bustling business district, whereas residential zones and office buildings are located within these station areas.

## Discussion

### Comparison of POI category vectors aggregation algorithms

In this study, we use an improved NLP-based framework to understand urban metro station areas. Considering how to integrate the POI semantic features to accurately represent urban areas, we improved the aggregation process of the existing framework. Therefore, to better comprehend the performance of the P-SIF model in aggregating station area vectors and describing their functional characteristics, we compared the P-SIF method with existing integration methods, including average summation, TF-IDF weighting, SIF, and P-SIF embedding. The averaging summation method, which is most commonly used in previous studies of urban functional region classification, computes the vectors for station areas by averaging vectors of POI category vectors within station areas. The TF–IDF method aggregates the POI category vectors that are weighted with TF-IDF scores. The SIF model weights the POI category vectors by a smooth inverse frequency $(a/(a+p(v)))$ and then removes the first principal component from the weighted average vectors to remove the common contexts. The remaining steps are the same as those described earlier, resulting in functional labels with different aggregation methods. The passenger flow data and the land use data of stations with different functions have their own characteristics, and they can reveal the functions of stations. Therefore, we use the travel characteristics and land use characteristics of each station as the input features of the model. We counted the proportions of different types of land use^45^ in the station area as the land use features. We use the proportion of the hourly incoming or outgoing passenger flow of each station to its total incoming or outgoing passenger flow on the day (including weekdays and weekends) as the travel feature. We used the features and labels obtained from the above methods to train a random forest model and compared their accuracy in terms of the mean absolute error (MAE), mean square error (MSE), and root mean square error (RMSE) scores. The random forest algorithm is a supervised learning algorithm that consists of multiple decision trees as an ensemble. The random forest algorithm has been extremely successful as a general-purpose classification and regression method because of its advantages in reducing the overfitting problem and handling high-dimensional features. The internal estimates are used to measure error, strength, correlation, and variable importance^46,47^. To increase the reliability of the results, we iterated the evaluation process 100 times and averaged the scores of the three evaluation indicators. In each iteration, the dataset was randomly split into a training set (70%), a validation set (10%), and a testing set (20%). Table 5 provides the error score results of these methods.

**Table 5:** Accuracy assessment with different aggregation methods.

Method	Land use feature			Travel feature		
	**MAE**	**MSE**	**RMSE**	**MAE**	**MSE**	**RMSE**
**Average**	7.8254	799.2813	27.1161	7.7472	792.8558	27.0775
**TF-IDF weighted**	9.0905	940.0272	29.2872	9.097	939.2574	29.3864
**SIF**	7.1193	719.7743	25.8448	7.1603	724.8487	26.0272
**P-SIF**	**7.0739**	**713.0572**	**25.7088**	**6.7168**	**499.7532**	**18.0208**

In the prediction process with the land use data or the travel data, the error scores of the P-SIF model were lower than those of the average method, the TF-IDF weighted method, and the SIF model. The results demonstrate that the P-SIF model provides a more precise description of the metro station area. However, the travel data and land data express the semantic features of the station area from different perspectives, and they may have incomplete consistency with the functional features we obtain on the basis of the POI configuration. However, the results still demonstrate that the P-SIF model has better performance for region representation.

The causes of station areas’ functional characteristics.

In the results, we understand the functional characteristics and spatial distribution of different station areas. Here we further discuss the reasons for its formation.

**(1) Mixing is the main characteristic of station area functional types.** As an important mode of transportation, metro serves people’s travel, metro station area has become a vital place to carry human activities. Accordingly, human activities affect metro station construction and show different functional characteristics. Due to the high human flow in the station areas in urban areas, the station areas in urban areas usually shows mixed functional characteristics and obvious commercial characteristics especially in urban area. The mixed functional types also show the main travel purpose of residents. For example, Group 0 (educational and residential mixed type) represents the purpose of travel for going to or leaving school. Group 4 (working and residential mixed type) represents the purpose of commuting.

**(2) working and residential are the most prominent functional types of the station area.** Working and living are the main themes of urban residents, which occupy most of the residents’ time. Commuting is the most important purpose for residents to travel. Therefore, the design of metro stations should connect the main residential and commercial areas of the city as much as possible. In the results, the 7 Groups all contain working and residential functional types, indicating the importance of commuting.

**(3) The Station area functional types difference between urban and suburban areas.** The population size difference is the reason of functional types difference. The station areas in the suburbs are usually based on the service of commuting, which is mostly manifested as a single function type. Commuting as the main way for residents to travel by metro. For example, Group 1 and Group 5 are station areas in suburbs and represent residential and working function type. The station areas in the urban serve more people, both residents and tourists. They are mostly mixed functional types. Like Group6, Group7 and Group8, urban areas usually undertake business, medical, intercity travels and other functional types in addition to basic commuting. So the functional types are mostly mixed. Tourism and commercial functional types also appear only in the station area within the urban area.

## Conclusion

This study introduces a framework for understanding the functional features of station areas on the basis of POI data and an embedding representation method, which improves the process of integrating POI semantic embeddings to accurately represent the station areas. In this framework, on the basis of the semantic vectors of POI categories trained by the GloVe model, the P-SIF model was employed to generate embedding representations of station areas. The P-SIF model can integrate the station area vectors with the topic characteristics and the importance information of POI categories, resulting in a more accurate representation. We further cluster the semantic vectors of the station areas via the AP algorithm and analyse the functional features of the station clusters via FET. In the results, 9 distinctive functional types are identified for urban metro station areas. The majority of metro stations have multiple types of urban functions. Most metro stations are characterized by residential functions. Another promising finding is that commercial functions account for a large proportion of Group 3, offering a reasonable implication for the correlation between metro stations and business. In addition, some patterns are revealed from the spatial distributions of station areas with different functions. The commercial stations have a more concentrated distribution and are mainly concentrated in the central area of the city, whereas the residential stations and working stations have a dispersed distribution in the city. We also observe that metro stations with geographical proximity may have similar functional patterns, which may be related to the fact that their station areas contain intersecting areas. These functional characteristics of the station areas we identified can provide assistance in assessing the development and functional complementarity of existing station areas.

There are also several limitations to the application of this framework. POIs are point data and do not have area properties, while areas can indicate importance to some extent. Therefore, describing the importance and impact of facilities within an area remains a great challenge. We hope to combine data from other platforms in future work to achieve metrics of influence and importance. Additionally, according to the spatial distribution characteristics of the stations, the density of stations in the central city is high and densely distributed, and the stations in the suburbs of the city are sparse. However, we defined a threshold of the same 15-min walkable range for all station areas so that a certain range of coverage exists for station areas located in the central city. Considering that the coverage areas should all fall within the influence areas of the involved stations, we do not segment the coverage area, leading to the possibility that stations in close proximity may have similar semantic features.

## Data Availability

The POI data and 15 min isochronous circles data analysed during the current study are available in the Gaode Open Platform [https://lbs.amap.com].
